# Editorial for the Special Issue Titled “Molecular Pharmacology and Interventions in Cardiovascular Disease”

**DOI:** 10.3390/ijms26178582

**Published:** 2025-09-03

**Authors:** Magdalena Zabielska-Kaczorowska

**Affiliations:** Department of Physiology, Medical University of Gdansk, 80-211 Gdansk, Poland; magdalena.zabielska@gumed.edu.pl; Tel.: +48-58-349-15-20

## 1. Introduction

Cardiovascular diseases (CVDs) include coronary artery disease, hypertension, and various cardiac disorders, among other conditions that impact the heart and vascular system. These diseases are commonly linked to a variety of risk factors, including diabetes, high blood pressure, and high cholesterol [1]. As the population’s mean age rises, so does the prevalence of cardiovascular disorders, which are intricate and multifaceted [2]. With a focus on drug delivery, controlled drug release, and targeting to the site of action to maximise the therapeutic effect, efforts are being made to develop novel pharmaceutical and non-pharmaceutical modulators in CVDs [3]. These advancements aim not only to enhance the efficacy of treatments but also to minimise side effects, ultimately improving patient outcomes. Researchers are exploring innovative approaches, including modern pharmacology and gene therapy, to further refine these delivery systems [4].

This special issue of the International Journal of Molecular Sciences (IJMS), entitled “Molecular Pharmacology and Interventions in Cardiovascular Disease”, contains 4 reviews and 6 original research papers written by a panel of experts who highlighted recent advances, discussed potential drug targets, and aimed to elucidate pharmacologic strategies that may be employed to promote cardiac repair after injury. These contributions provide valuable insights into the intricate mechanisms underlying cardiovascular pathology and offer a glimpse into future therapeutic avenues. The collective findings underscore the importance of continued research in this critical area of medical science to achieve therapeutic gain and enhance the efficacy of protocols for cardiac therapy.

## 2. Research

The original articles cover a range of topics from the use of novel inhibitors to analyses of vascular permeability mediators in cardiovascular disorders. Two publications are focused on the treatment of an aneurysm. Increased levels of matrix metalloproteinases (MMPs) and destabilisation of the vessel wall due to the breakdown of the extracellular matrix’s (ECM) structural elements, primarily collagen and elastin, are caused by accelerated inflammatory processes during aneurysm development. ECM proteolysis is inhibited by tissue inhibitors of metalloproteinases (TIMPs), which directly control MMP activity. Here, Golombek et al. suppressed MMP-9 by synthetic TIMP-1 encoding mRNA exogenously delivered into the aorta, which triggered the expression of TIMP-1 protein. These findings suggested that TIMP-1 mRNA administration is promising as an aneurysm therapy method [5]. In order to prevent aneurysm formation in Marfan syndrome (MFS), strategies to block downstream effectors of angiotensin II type 1 receptor (AT1R)-mediated signalling were investigated in the work of Callejon et al. It was found that inhibiting the progression of aneurysms in MFS may require total blocking of AT1R function, which can be accomplished by administering a high dosage of losartan [6]. The next set of two investigations is related to the inflammation strongly involved in cardiovascular disease. Inhibition of the interleukin-6 receptor (IL-6R) has demonstrated efficacy in alleviating myocardial damage and decreasing the levels of the prothrombotic and inflammatory mediator, neutrophil extracellular traps (NETs). A major contributor to the development of NET consists of the enzyme peptidylarginine deiminase 4 (PAD4). In STEMI patients, a correlation between PAD4, IL-6R, and troponin release was shown by Kindberg et al. [7]. The pathophysiology of atherosclerosis and the development of in-stent restenosis (ISR) are significantly influenced by inflammation. It was found by Hytönen et al. that a novel synthetic flavonoid, 3,7-dihydroxy-isoflav-3-ene (DHIF) showed promise in limiting ISR through antioxidant action. DHIF treatment did not postpone endothelial healing following stenting injury and markedly decreased inflammation and proliferation in the restenotic lesions [8]. Investigation of Luecht et al. was conducted into the inflammatory response in macrophages triggered by perioperative serum samples taken from congenital heart defect (CHD) patients undergoing cardiopulmonary bypass (CPB) heart surgery. By activating STAT3 through IL-6 and IL-8, CPB triggered an increase in the production of cytokines and mediators of vascular permeability. Treating those patients with Stattic increased TNFα expression while attenuating all mediators examined [9]. In the work of Brosinsky et al. using the inducible cardiomyocyte-specific monoamine oxidase B (cmMAO-B) knockout mouse model, the effects of ROS generation mediated by MAO-B on RV dilatation and function in response to pulmonary artery banding were examined [10]. During pulmonary hypertension (PH), the elevated production of reactive oxygen species (ROS) in the mitochondria is crucial for the development of right ventricular hypertrophy (RVH) and failure (RVF). Compared to their littermates, cmMAO-B KO mice were shielded against RV dilatation, hypertrophy, and dysfunction after RV pressure overload. The hypothesis that cmMAO-B plays a significant role in RV hypertrophy and failure during PH was validated by these findings.

## 3. Review

Review articles present several up-to-date aspects of the pharmacological interventions in CVDs. Kulpa et al., reviewed data showing that research on catestatin (CTS) is encouraging and suggests that it may be used to treat a variety of CVDs. It was found that CTS stimulates endothelial cells and cardiomyocytes to produce more NO. Additionally, for patients with mildly decreased or retained HF, CTS may serve as a biochemical marker. However, there are some conditions, including atherosclerosis, where studies regarding the impact of CTS on vessel wall remodelling were conflicting, and its significance in these conditions is still unclear [11]. Liu et al. made an insightful review about the therapeutic strategies of cirrhotic cardiomyopathy (CCM). Patients with CCM may benefit from treatments including antioxidants, anti-inflammatory, and anti-apoptotic drugs. Such strategies are now primarily restricted to animal studies, hence carefully planned clinical trials are required to validate these compounds. Another possible therapeutic agent are non-selective beta-blockers (NSBBs), which should, in theory, have therapeutic effects on CCM. Nevertheless, there is inconsistency among the outcomes from various studies [12]. Menezes et al. summarised the current state of treatment for hypertrophic cardiomyopathy (HCM), a complex heart disease characterised by alterations in a number of biological processes, such as ROS control, ionic homeostasis, structural remodelling, and metabolic balance. It was proposed that mitochondrial redox signalling and genetic mutations are the key regulating variables in HCM [13]. Szczepanska-Sadowska et al. explored the interactions between the hormones of the hypothalamo–pituitary–adrenal axis and vasopressin (AVP). This review summarises recent knowledge about steroid hormones and AVP that are often released together and act closely together to control behaviour, metabolism, blood pressure, and water-electrolyte balance. Significant changes occur in the interactions between AVP and HPA during inflammation, neurogenic stress, and metabolic, respiratory, and cardiovascular disorders. In metabolic diseases, inappropriate interactions between AVP and steroids may cause or accelerate cardiovascular diseases [14].

This Special Issue underlines the richness but also the complexity of approaches in CVDs treatment. The present challenge is to accelerate the exploration of unique pharmacological targets for clinical applications. As the Guest Editor, I hope that the findings included in this Special Issue will inspire further investigations in this challenging field.

## References

[1] Prousi G.S., Joshi A.M., Atti V., Addison D., Brown S.A., Guha A., Patel B. (2023). Vascular Inflammation, Cancer, and Cardiovascular Diseases. Curr. Oncol. Rep..

[2] Gaidai O., Cao Y., Loginov S. (2023). Global Cardiovascular Diseases Death Rate Prediction. Curr. Probl. Cardiol..

[3] Stern C.S., Lebowitz J. (2010). Latest Drug Developments in the Field of Cardiovascular Disease. Int. J. Angiol..

[4] Plowright A.T., Engkvist O., Gill A., Knerr L., Wang Q.D. (2014). Heart Regeneration: Opportunities and Challenges for Drug Discovery with Novel Chemical and Therapeutic Methods or Agents. Angew. Chem.—Int. Ed..

[5] Golombek S., Doll I., Kaufmann L., Lescan M., Schlensak C., Avci-Adali M. (2024). A Novel Strategy for the Treatment of Aneurysms: Inhibition of MMP-9 Activity through the Delivery of TIMP-1 Encoding Synthetic MRNA into Arteries. Int. J. Mol. Sci..

[6] Valdivia Callejon I., Buccioli L., Bastianen J., Schippers J., Verstraeten A., Luyckx I., Peeters S., Danser A.H.J., Van Kimmenade R.R.J., Meester J. (2024). Investigation of Strategies to Block Downstream Effectors of AT1R-Mediated Signalling to Prevent Aneurysm Formation in Marfan Syndrome. Int. J. Mol. Sci..

[7] Kindberg K.M., Nordeng J., Langseth M.S., Schandiz H., Roald B., Solheim S., Seljeflot I., Stokke M.K., Helseth R. (2025). IL-6R Signaling Is Associated with PAD4 and Neutrophil Extracellular Trap Formation in Patients with STEMI. Int. J. Mol. Sci..

[8] Hytönen J.P., Leppänen O., Taavitsainen J., Ylä-Herttuala S. (2024). Synthetic Flavonoid 3,7-Dihydroxy-Isoflav-3-Ene (DHIF) Reduces In-Stent Restenosis in an Atherosclerotic Watanabe Heritable Hyperlipidemic Rabbit Stent Model. Int. J. Mol. Sci..

[9] Luecht J., Pauli C., Seiler R., Herre A.L., Brankova L., Berger F., Schmitt K.R.L., Tong G. (2024). Prolonged Extracorporeal Circulation Leads to Inflammation and Higher Expression of Mediators of Vascular Permeability Through Activation of STAT3 Signaling Pathway in Macrophages. Int. J. Mol. Sci..

[10] Brosinsky P., Heger J., Sydykov A., Weiss A., Klatt S., Czech L., Kraut S., Schermuly R.T., Schlüter K.D., Schulz R. (2024). Does Cell-Type-Specific Silencing of Monoamine Oxidase B Interfere with the Development of Right Ventricle (RV) Hypertrophy or Right Ventricle Failure in Pulmonary Hypertension?. Int. J. Mol. Sci..

[11] Kulpa J., Paduch J., Szczepanik M., Gorący-Rosik A., Rosik J., Tchórz M., Pawlik A., Gorący J. (2025). Catestatin in Cardiovascular Diseases. Int. J. Mol. Sci..

[12] Liu H., Ryu D., Hwang S., Lee S.S. (2024). Therapies for Cirrhotic Cardiomyopathy: Current Perspectives and Future Possibilities. Int. J. Mol. Sci..

[13] da Menezes Junior A.S., de França-e-Silva A.L.G., de Oliveira H.L., de Lima K.B.A., de Porto I.O.P., Pedroso T.M.A., de Silva D.M.E., Freitas A.F. (2024). Genetic Mutations and Mitochondrial Redox Signaling as Modulating Factors in Hypertrophic Cardiomyopathy: A Scoping Review. Int. J. Mol. Sci..

[14] Szczepanska-Sadowska E., Czarzasta K., Bogacki-Rychlik W., Kowara M. (2024). The Interaction of Vasopressin with Hormones of the Hypothalamo–Pituitary–Adrenal Axis: The Significance for Therapeutic Strategies in Cardiovascular and Metabolic Diseases. Int. J. Mol. Sci..

